# T140 blocks the SDF-1/CXCR4 signaling pathway and prevents cartilage degeneration in an osteoarthritis disease model

**DOI:** 10.1371/journal.pone.0176048

**Published:** 2017-04-20

**Authors:** Kun Wang, Yanlin Li, Rui Han, Guofeng Cai, Chuan He, Guoliang Wang, Di Jia

**Affiliations:** 1 Department of Sports Medicine, First Affiliated Hospital of Kunming Medical University, Kunming, Yunnan, China; 2 Department of Diabetology, First Affiliated Hospital of Kunming Medical University, Kunming, China; Universite de Lyon, FRANCE

## Abstract

Osteoarthritis (OA) is one of the most common diseases affecting older people; however, there remains no effective targeted drug to combat OA. The aims of this study were (1) to explore the effect of T140 in regulating degeneration of articular cartilage in vivo by targeted blocking of the SDF-1/CXCR4 signaling pathway, and (2) to provide experimental evidence for the development of a novel OA-targeted pharmacotherapy. Thirty-six healthy Hartley guinea pigs were randomly divided into three groups: a T140-treated group (n = 12), a phosphate buffer saline control group (n = 12) and an untreated control group (n = 12). At 2, 4, 6, 8, 10 and 12 weeks of treatment, SDF-1 in serum was quantified by enzyme-linked immunosorbent assay. At 12 weeks of treatment, the cartilage from knee tibial plateau in the knee joint was collected for H&E, Safranin-O staining and Mankin grading; measurement for mRNA levels of matrix metalloproteinases (MMP-3, MMP-9 and MMP-13), aggrecan (ACAN) and collagen II (Col II) using RT-PCR; and measurement for Col II protein levels by western blot. Results showed that SDF-1 in serum increased in the T140 group and increased in the control groups. H&E and Safranin-O staining revealed less cartilage loss in T140-treated animals compared to controls. The mRNA levels of MMP-3, MMP-9 and MMP-13 in cartilage were much lower in the T140 group than other groups, but mRNA levels of ACAN and Col II in cartilage were higher in the T140-treated group. Col II protein levels in the T140 group and control groups were different. T140 can downregulate the expression of matrix-degrading enzyme and lessen the degeneration of cartilage by blocking the SDF-1/CRCR4 signaling pathway in vivo. This mechanism may present a pharmacological target for the treatment of OA.

## Introduction

Osteoarthritis (OA) is one of the most common diseases affecting older people[1,2]. Non-surgical treatments are usually first line management of OA-associated knee degeneration[3], but there are no targeted drugs to combat OA because of its elusive pathogenic mechanism[4] and research into novel pharmacological interventions remains critical[5].

Recently it was shown that the SDF- 1/CXCR4 signaling pathway plays a key role in the pathogenesis of cartilage degeneration[6,7]. In OA, synovial cells in joints synthesize and secrete stromal cell derived factor-1 (SDF-1) into the synovial fluid[8] and chondrocytes in cartilage tissue express chemokine receptor 4 (CXCR4)–a major receptor of SDF-1. Once SDF-1 binds to CXCR4 it induces chondrocytes to release various enzymes causing cartilage degradation and increasing the pathological process of OA[9,10]. Here, we explored the efficacy of CXCR4 antagonists in delaying the degeneration of articular cartilage by blocking the SDF-1/CXCR4 signaling pathway.

Recent evidence suggests that a small peptide named T140 is a complete CXCR4 inhibitor and can totally block the SDF-1/CXCR4 signaling pathway[11]. T140 is currently used as a targeted drug in cancer therapy and anti-HIV research[12,13], but has not been tested in OA disease models. We hypothesized that T140 may alleviate the cartilage degeneration seen in OA and designed this study to explore the role of a T140-blocked SDF-1/CXCR4 signaling pathway and OA cartilage degeneration attenuation in vivo.

## Materials and methods

### Animals and groups

Hartley guinea pigs develop spontaneous knee OA at around age 9 months of age[4]. Thirty-six male Hartley guinea pigs (9-months-old, weight = 600±50 g) were obtained from the Institute of Zoology, Chinese Academy of Sciences (Kunming, China). All guinea pigs were fed in tray type cages with sawdust as bedding material; sawdust was replaced every day to keep the living environment dry. Every four guinea-pig were housed and fed in one cage. Clean water in a lick type bottle was supplied. The breeding room was kept quiet to avoid frightening animals. A light and dark cycle was formed by turning on the sunlight lamp at 08.00 h and off at 20.00 h. All animal treatment and care was approved by the Kunming Medical University Animal Care Committee.

Thirty-six male Hartley guinea pigs were randomly divided into three groups. Group A animals (T140-treated group, n = 12) were treated with T140 via a constant infusion osmotic Mini-pump (Alza Corporation, Mountain View, USA). T140 lyophilized powder (Biopeptide, San Diego, USA) was diluted in phosphate buffer saline (PBS) solution and pumped into subcutaneous tissue at a concentration of 180 ug/d. Group B animals (PBS control group, n = 12) received PBS via constant infusion osmotic Mini-pump. Group C (untreated OA control, n = 12) animals was left untreated as a blank control group.

### Mini-osmotic pump implantation and surgical procedures

Guinea pigs were anesthetized with ketamine (50 mg/kg body weight) and xylazine (5 mg/kg body weight). An area (~4 cm^2^) of the back over the dorsolateral thorax in Group A and B animals was clipped and prepared for aseptic surgery. After disinfection using 75% alcohol, the skin was cut a small incision (~1 cm) and a small subcutaneous pocket was formed by blunt dissection. A Mini-osmotic pump was embedded into this pocket. Before insertion, each pump was filled into 200 ul of solution containing either 50 mg/ml T140 in PBS (Group A) or PBS alone (Group B). At an average pumping rate of 0.15 μl/per hour, each animal in Group A received 180 μg of T140 per day. The pumps were exchanged once after six weeks from the date of implantation.

### Sample harvest

At 2, 4, 6, 8, 10 and 12 weeks of treatment, 5∼8 animals in each group were randomly chosen for blood collection via cardiac puncture. Blood samples were centrifuged for 5∼10 min (3000 r/min) to remove cells and debris within 1 h, and supernatants were sterilized using a 0.22 μm Millipore filter, and stored in a -80°C cryogenic refrigerator in order to later detect SDF-1 content using enzyme-linked immunosorbent assay (ELISA). At 12 weeks all guinea pigs were sacrificed by injecting pentobarbital sodium (30 mg/kg) into the peritoneal cavity. Cartilages from the tibial plateau in each group were harvested for histological assay, PCR testing and western blot analysis.

### ELISA

Serum SDF-1 in each group was analyzed using an ELISA kit (R&D Systems, Minneapolis, USA) according to the manufacturer’s protocol. ELISA assays were performed in duplicate wells for each sample. Optical density values in developed plates were determined using a microplate reader set to 450 nm, then; depending on linear regression equation standards we calculated the concentration of SDF-1 in samples.

### Histological observation

Gross morphologic lesions on the tibia plateau were visualized with hematoxylin and eosin (H&E) and Safranin-O staining. In short, cartilage tissue specimens from the tibial plateau were fixed with 10% formalin solution for 24 h. Following gradient ethanol dehydration and paraffin embedding, specimens were cut into 5 μm sections for H&E and Safranin-O staining to observe degeneration and the distribution of chondrocytes. The grade of proteoglycan and cartilage degradation was quantified with the modified Mankin grading system[14]. Photographs were taken using a Nikon microscope. Three clear images from the tibial plateau at regular intervals in each animal were selected to analyze Mankin histological scoring criteria and cartilage degradation.

### Reverse transcription-polymerase chain reaction (RT-PCR) assay

Total RNA was isolated from tibial plateau cartilage tissue with an RNeasy isolation kit (Fermentas, Burlington, Canada) and the quantity and purity of extracted RNA was evaluated with a spectrophotometer. 1 μg of total RNA was transcribed into cDNA according to RevertAid^TM^ First Strand cDNA Synthesis Kit instructions (Fermentas, Burlington, Canada), and 40 ng/μl of this obtained cDNA was used as a template for PCR amplification in order to quantify the relative content of each mRNA using a LightCycler480 SYBR Green I Master (Roche Diagnostics, Mannheim, Germany). β-Actin was used as an internal reference. Primers for β-Actin, MMP-3, MMP-9, MMP-13, ACAN and Col II designed by OMIGA v2.0 (Genetics Computer Group, Madison, USA) were used to amplify products contained within the coding sequence of the various RNAs. Expression levels were measured in triplicate. Primer sequences are listed in Table 1. The data are given as a threshold cycle (Ct). Fold changes in gene expression were calculated as 2^-Δ(ΔCt)^. The comparative Ct (threshold cycles) method was used to evaluate the expression level of each target gene relative to the level in the untreated group.

**Table 1:** Sequences of primers used for RT-PCR.

Gene	Upstream primer 5 '-3'	Downstream primer 5 '-3'	Length (bp)
β-Actin	CCACCATGTACCCAGGCATT	ACTCCTGCTTGCTGATCCAC	177
MMP-3	GGACAAAGGATACAACAGGGAC	TCATCTTGAGACAGGCGAAA	157
MMP-9	AGGAAAGGCGCTGCTCTTCA	GGAGAACACATGGTCCACCG	103
MMP-9	CGCTACCTGAAATCATACTACCA	CCTGTCACCTCTAAGCCAAAG	122
ACAN	ACATCTCAGCAGCATCATCACC	CATCACCACGCAGTCCTCAC	199
Col Ⅱ	ATGCACCTTGGATGCCATGA	ATGCACCTTGGATGCCATGA	103

### Western blot

100 mg of frozen cartilage tissue was dissected and immediately transferred to the RIPA buffer with a protease inhibitor cocktail, and then homogenized on ice using the SilentCrusher M Homogenizer. The homogenate was centrifuged (12000 RPM for 10 min at 4°C). The supernatant was collected and quantified using a BAC Protein Assay Kit (Pierce, Rockford, USA). In brief,10 μg of total protein was electrophoresed in 10% SDS-PAGE, and proteins from sodium dodecyl sulfate (SDS) gels were electrophoretically transferred to a polyvinylidene difluoride membrane (Millipore, Bedford, USA) and blocked with 5% nonfat milk in Tris-buffered saline-Tween 20 (TBS-T). Membranes were probed by a human anti-Col Ⅱ monoclonal antibody (1:1000 dilution; Proteintech Group, Chicago, USA) and anti-β-actin polyclonal antibody (1:1000 dilution; Cell Signaling Technology, Danvers, USA). Horseradish peroxidase-conjugated goat anti-rabbit immunoglobulin G (IgG) (H+L) (1:3000 dilution, Bio-Rad Laboratories, Richmond, USA) was used as the secondary antibody. Chemiluminescence was visualized by a Tanon-4500 digital image processing system (Tanon, Shanghai, China) and light was then detected by photographic film.

### Statistical analysis

Statistical analysis was performed using SPSS v19.0 (SPSS Inc, Chicago, USA). All data are presented as mean ± standard deviation (SD). ANOVA for repeated measurement data and LSD-t test were used for statistical analysis. P values < 0.05 were considered statistically significant.

## Results

### T140 decreased SDF-1 in serum

**Fig 1:**
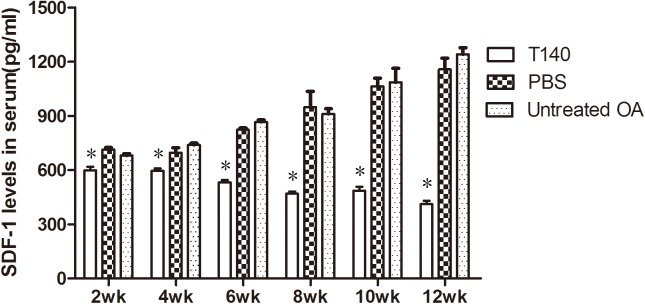
Levels of SDF-1 in the serum of guinea pigs in three experimental groups measured using ELISA (n = 5). T140: Hartley guinea pigs treated with T140. PBS: control group treated with saline. Untreated OA: untreated control Hartley guinea pigs. Data are expressed as mean ± SD. *P < 0.05 compared with PBS control group and untreated control group.

Levels of SDF-1 in serum in the T140-treated group decreased gradually as the length of treatment increased, but increased over the course of the experiment in the PBS and blank control groups. SDF-1 levels in the T140 group were different to both control groups at any time point in our experiment (P < 0.05); SDF-1 levels in the PBS control group and blank control group were not different (P > 0.05). This pattern indicates that T140 could reduce levels of SDF-1 in serum (Fig 1). The detailed data were shown in (S1 Table).

### T140 attenuated the severity of OA cartilage damage in vivo

**Fig 2:**
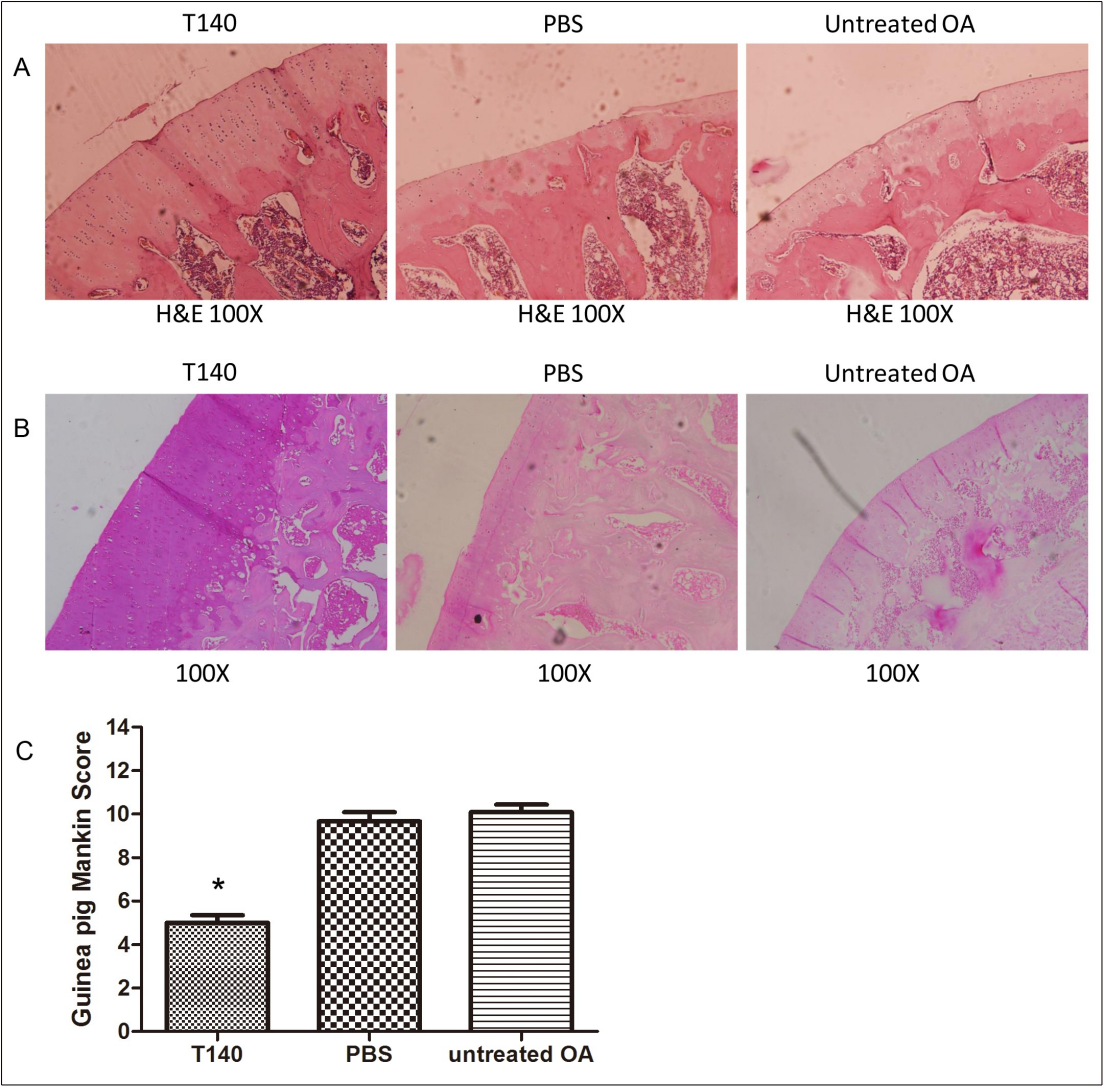
T140 alleviated OA cartilage degeneration. (A) H&E staining showing that cartilage degeneration in the T140 group is reduced compared to a PBS control group and untreated control group. (B) Safranin-O staining of cartilage tissue in each group demonstrates (C) lower Mankin scores in the T140 group compared to controls. * P < 0.05 compared with each control group.

Our morphological results showed that severe OA cartilage lesions were observed in the PBS control group and blank control group, however, only minor OA changes were observed in the T140-treated group (Fig 2A and 2B). The basic structure of articular cartilage tissue in the T140-treated group was smoother, hypercellular and contained more trabecular bone, less empty lacunae and no fibrosis when compared with control groups. Modified Mankin scores in the PBS control group (9.67±1.44) and untreated control group (10.08±1.24) indicated similar severe degeneration (P = 0.455) (Fig 2C). The detailed data in each group were shown in (S2 Table).Less cartilage damage was observed in the T140-treated group with a modified Mankin score of 5.00±1.21 (P < 0.01) when compared with both control groups.

### T140 downregulated the expression of MMP-3, MMP-9 and MMP-13 and upregulated the expression of Col II and aggrecan

**Fig 3:**
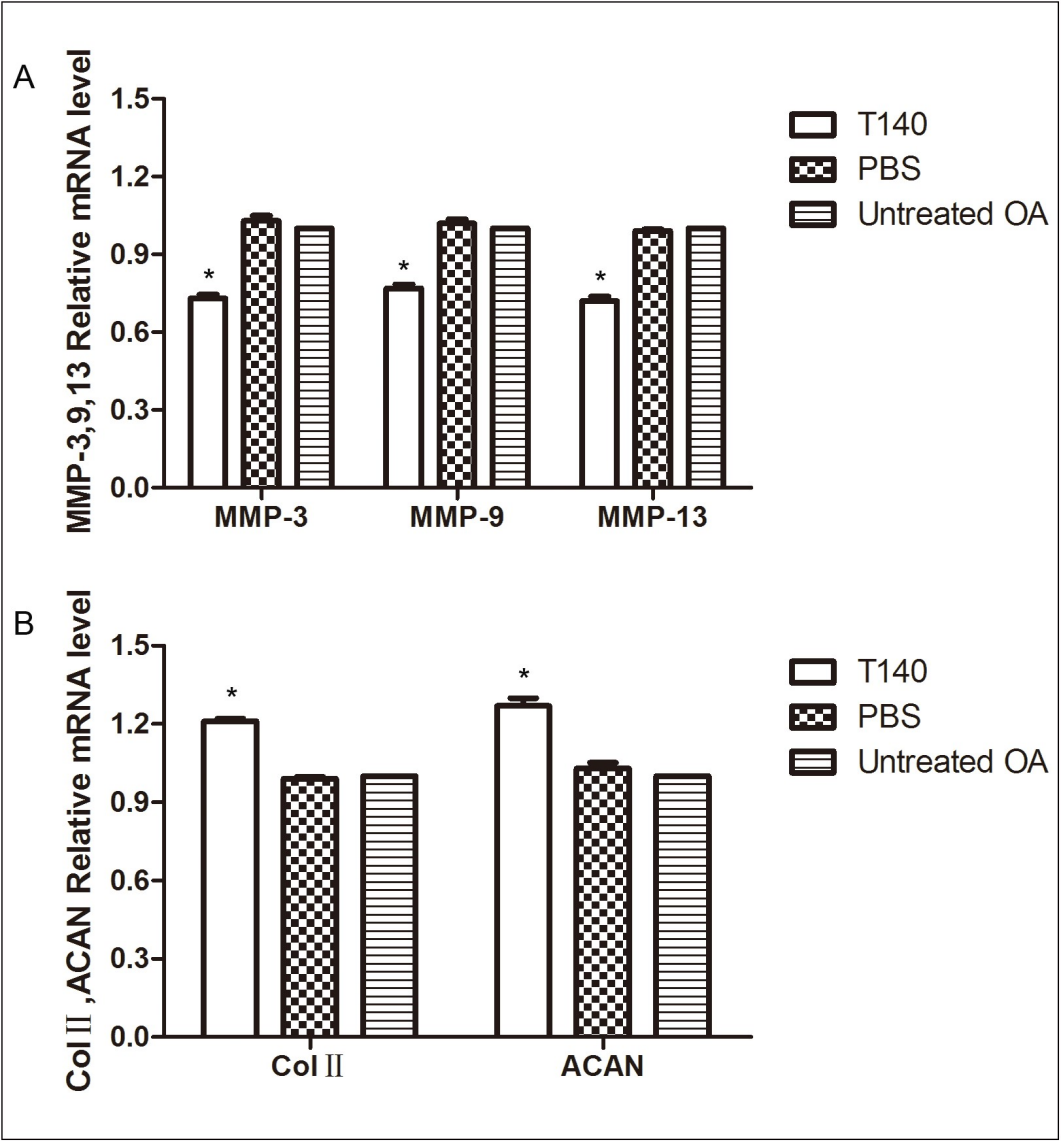
The expression of MMP-3, MMP-9, MMP-13, Col II and aggrecan measured using RT-PCR. (A) The mRNA expression of MMP-3, MMP-9 and MMP-13 was lower in the T140 treatment group compared with control. (B) The expression of ColⅡ and aggrecan was higher in the T140 treatment group compared with controls. T140: animals treated with T140. PBS: control group treated with saline. Untreated OA: untreated control Hartley guinea pigs. *P < 0.05 compared with each control group.

To investigate the effect of T140 on cartilage degeneration, we examined the expression patterns of the cartilage-degrading proteases MMP-3, MMP-9 and MMP-13, and the cartilage matrix proteins Col II and aggrecan in cartilage. We found that mRNA expression patterns for MMP-3, MMP-9 and MMP-13 in the T140 group were lower than that in the PBS control group and blank control group. However, the mRNA relative expressions of Col II and aggrecan were higher in the T140 group than the other groups. The mRNA expression patterns of these five genes in the T140 group compared with those in the other groups were statistically different (P < 0.05) (Fig 3A and 3B). The detailed mRNA levels in each group were shown in (S3 Table).

### T140 reduced degradation of type II collagen in the cartilage matrix

**Fig 4:**
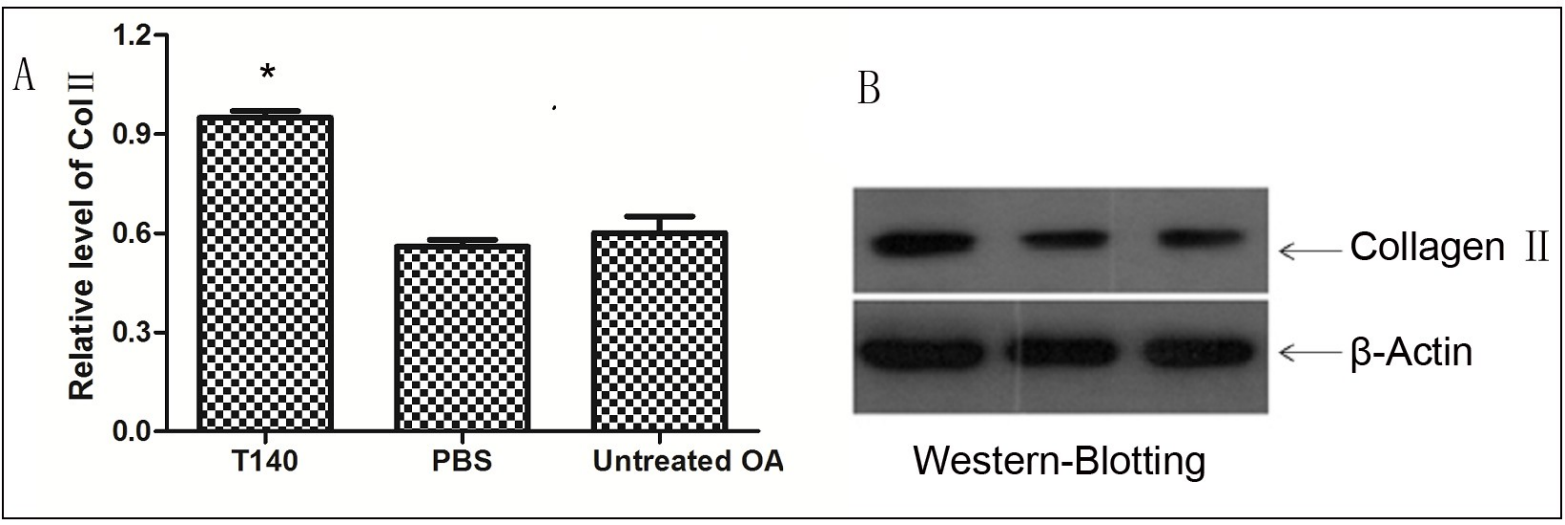
T140 reduced degradation of type II collagen in the cartilage matrix. (A) Western blotting showed that T140 increased protein levels of Col II. The high concentration of Col II in T140-treated groups was observed in the Hartley guinea pigs treated with T140 (4A, 4B)

Given that type II collagen was the main component of the cartilage matrix, we determined the effects of T140 on type II collagen. We found that type II collagen protein levels in the T140-treated group were higher than in the blank control group and PBS control group. Col II protein levels in the T140 group differed from levels measured in the blank and PBS control groups (P <0.05); Col II protein levels in the PBS control group and blank control group did not differ (P = 0.402) (Fig 4). The detailed protein levels in each group were shown in (S4 Table).

## Discussion

OA is a common chronic disease that causes articular cartilage degeneration[15]. It often results in disability in patients with advanced OA and surgical treatments are required in order to reduce pain and restore joint function. It is crucial to prevent the development of OA during early stages of the disease in order to reduce the incidence of disability.

Although the aetiological mechanisms of OA remain unclear, studies suggest that the SDF-1/CXCR4 signaling pathway plays a vital role in its development[6,7,16,17]. In the knee, SDF-1 is produced by the synovium and secreted into the joint cavity and blood, however, its receptor CXCR4 is expressed on chondrocytes in the joint. Once SDF-1 binds to CXCR4 it activates extracellular signal-regulated enzyme (Erk) and associated kinase (p38 MAP kinase) signaling pathways and induces chondrocytes to release matrix metalloproteinases–the main enzymes that degrade cartilage matrix and that drive the pathogenesis of OA[18,19].

Previous studies showed that T140 was a low cytotoxic antagonist of the CXCR4 receptor and could effectively block the SDF-1/CXCR4 signaling pathway[20,21]. T140 was mainly used as a targeted drug against chronic inflammatory diseases, AIDS and tumors[12,13,22,23]. Here, we found slight OA changes in T140 treated animals. More lesions appeared in the PBS control group and untreated control group including cartilage softening, sparsity of bone trabecula, cartilage structural confusion, increased chondrocyte numbers and empty lacunae. Mankin scores for cartilage were lower in the treatment group compared to the control groups, indicating that T140 reduces cartilage degeneration.

Recent studies have showed that SDF-1 is considered a crucial cytokine causing OA and is likely associated with the progression of inflammation[4,24]. SDF-1 signaling blockade with a unique drug will result in more benefits in the treatment of OA over other drugs[25]. Studies have found that SDF-1 secreted from the synovium and bone marrow, especially when stimulated by inflammatory factors, integrates with CXCR4 and can trigger the SDF-1/CXCR4 signaling pathway to induce cartilage matrix degradation. SDF-1 levels in the plasma and synovial fluid are closely related with the severity of OA and may serve as an effective biomarker of the disease[26]. Previous study showed that the level of SDF-1 in serum declined after synovectomy[10]. The level of SDF-1 in the synovial fluid could be reduced by a CXCR4 antagonist called AMD3100 [4]. In our investigation, elevated serum SDF-1 was observed in a guinea pig OA model and this pathological increase in serum SDF-1 could be inhibited by T140. We speculate that T140 as a CXCR4 antagonist can reduce the level of SDF-1 in synovial fluid by inhibiting the secretions of synovial cells, which reduces the level of SDF-1 in serum.

The imbalance between destructive and protective factors are considered important during articular cartilage degeneration[27]. MMP family members, such as MMP-3, MMP-9 and MMP-13, are the main disruptive factors in cartilage degeneration[28,29,30]. These proteins cause the development of OA by degrading collagen and disrupting the structure of proteoglycan in the cartilage matrix[31]. In contrast, collagen II and aggrecan are considered articular cartilage protective factors and play an important role in maintaining the elasticity and hardness of cartilage[32]. Loss of aggrecan is believed to be the main manifestation of early arthritis. Type II collagen and aggrecan are the main components of the cartilage matrix[33], and their expression and apoptosis are the best indicators of cartilage degeneration. In our study, mRNA expression of MMP-3, MMP-9 and MMP-13 in the T140-treatment group was lower than in the untreated control group and PBS control group; and mRNA expression levels of collagenⅡand aggrecan were higher in the T140-treated group. This finding demonstrates that T140 can block SDF-1 and CXCR4 binding and eliminate the stimulatory trigger for the synthesis of MMPs and reduce the degeneration of articular cartilage. T140 can be used as a targeted antagonist of CXCR4 in the prevention of cartilage degeneration.

To our knowledge, this study is the first to indicate that T140 blocks the SDF-1/CXCR4 signaling pathway in vivo, reduces mRNA expression levels of MMP-3, MMP-9 and MMP-13, slows the degradation of type II collagen and aggrecan in cartilage tissue, and lessens the degeneration of cartilage tissue in an OA disease model. Further work will involve repeating this study in mini-pig or monkey to verify the attenuation of OA by T140.

## Supporting information

S1 Table Levels of SDF-1 in the serum of guinea pigs in three groups measured using ELISA (pg/ml). The data were corresponded to Fig 1.
(PDF)

S2 Table Mankin scores in each group. The data were corresponded to Fig 2.
(PDF)

S3 Table The expression of MMP-3, MMP-9, MMP-13, Col II and ACAN measured using RT-PCR. The data were corresponded to Fig 3.
(PDF)

S4 Table T140 reduced degradation of type II collagen in the cartilage matrix measured using Western blot. The data were corresponded to Fig 4.
(PDF)
