# Supplementary material for: T140 blocks the SDF-1/CXCR4 signaling pathway and prevents cartilage degeneration in an osteoarthritis disease model
Source: PLoS One. 2017 Apr 20;12(4):e0176048. doi: 10.1371/journal.pone.0176048 (PMC5398617; doi:10.1371/journal.pone.0176048)
Supplement: S1 Table — The data were corresponded to Fig 1. (PDF) [file pone.0176048.s001.pdf]

S1 Table. Levels of SDF-1 in the serum of guinea pigs in three groups measured using ELISA (pg/ml)

Time	Groups			F value	P value
	T140 group	PBS group	Untreated group		
2w	598.87±42.94	713.12±24.96	680.27±23.10	17.30	0.00
4w	596.56±29.79	696.26±61.38	738.61±23.94	15.26	0.00
6w	531.63±30.17	822.91±23.49	866.37±25.05	237.81	0.00
8w	468.76±28.05	948.43±194.17	910.92±62.60	25.17	0.00
10w	484.87±51.05	1062.78±103.81	1085.81±173.41	40.02	0.00
12w	411.27±41.94	1157.41±139.14	1240.43±80.78	113.14	0.00
