# Supplementary material for: T140 blocks the SDF-1/CXCR4 signaling pathway and prevents cartilage degeneration in an osteoarthritis disease model
Source: PLoS One. 2017 Apr 20;12(4):e0176048. doi: 10.1371/journal.pone.0176048 (PMC5398617; doi:10.1371/journal.pone.0176048)
Supplement: S2 Table — The data were corresponded to Fig 2. (PDF) [file pone.0176048.s002.pdf]

S2 Table. Mankin scores in each group

Groups	Mankin scores	F value	P value
T140 group	5.00 ± 1.21	56.75	0.00
PBS group	9.67 ±1.44		
Untreated group	10.08 ±1.24		
