# Supplementary material for: T140 blocks the SDF-1/CXCR4 signaling pathway and prevents cartilage degeneration in an osteoarthritis disease model
Source: PLoS One. 2017 Apr 20;12(4):e0176048. doi: 10.1371/journal.pone.0176048 (PMC5398617; doi:10.1371/journal.pone.0176048)
Supplement: S3 Table — The data were corresponded to Fig 3. (PDF) [file pone.0176048.s003.pdf]

S3 Table: The expression of MMP-3, MMP-9, MMP-13, Col II and ACAN measured using RT-PCR.

gene	T140 group	PBS group	Untreated group	F value	P value
MMP-3	0.73±0.01	1.003±0.02	1.00±0.00	1256.52	0.00
MMP-9	0.77±0.01	1.02±0. 01	1.00±0.00	579.23	0.00
MMP13	0.72±0.01	0.99±0.01	1.00±0.00	931.51	0.00
ColII	1.21±0.01	0.99±0. 01	1.00±0.00	875.40	0.00
ACAN	1.27±0.03	1.03±0.02	1.00±0.00	216.92	0.00
