# Supplementary material for: T140 blocks the SDF-1/CXCR4 signaling pathway and prevents cartilage degeneration in an osteoarthritis disease model
Source: PLoS One. 2017 Apr 20;12(4):e0176048. doi: 10.1371/journal.pone.0176048 (PMC5398617; doi:10.1371/journal.pone.0176048)
Supplement: S4 Table — The data were corresponded to Fig 4. (PDF) [file pone.0176048.s004.pdf]

S4 Table: T140 reduced degradation of type II collagen in the cartilage matrix measured using Western blot

groups	Relative level of Col II	F value	P value
T140 group	0. 95±0.02	170.16	0.00
PBS group	0.56±0.02		
Untreated group	0.60±0.05		
